# The fine specificity of IgM anti-citrullinated protein antibodies (ACPA) is different from that of IgG ACPA

**DOI:** 10.1186/ar3524

**Published:** 2011-11-30

**Authors:** Parawee Suwannalai, Annemiek Willemze, Linda van Toorn, Gerrie Stoeken-Rijsbergen, Nivine Levarht, Jan Wouter Drijfhout, Tom W J Huizinga, Rene E M Toes, Leendert A Trouw

**Affiliations:** 1 Department of Rheumatology, Leiden University Medical Center (LUMC), PO Box 9600, NL-2300 RC Leiden, The Netherlands; 2 Department of Immunohematology and Blood Tranfusion, PO Box 9600, NL-2300 RC Leiden University Medical Center (LUMC), Leiden, The Netherlands

## Abstract

**Introduction:**

The antigen recognition pattern of immunoglobulin M (IgM) could, when directed against protein antigens, provide an indication of the antigenic moieties triggering new B cells. The half-life of IgM is short and memory B cells against T-cell-dependent protein antigens typically produce IgG and not IgM antibodies. In this study, we analyzed whether a difference exists between the fine specificity of IgM versus IgG anti-citrullinated protein antibodies (ACPAs).

**Methods:**

We determined the fine specificity of IgM and IgG ACPAs in 113 ACPA-positive rheumatoid arthritis patients with IgM cyclic citrullinated peptide 2 (CCP2) levels above 100 AU/ml. Fine specificity was assessed by performing ELISA using citrullinated peptides derived from vimentin, fibrinogen-β, fibrinogen-α and α-enolase, as well as citrullinated proteins fibrinogen and myelin basic protein. The arginine counterparts were used as controls.

**Results:**

Recognition of defined citrullinated antigens by IgM ACPA was confined to samples that also displayed recognition by IgG ACPA. However, the IgM ACPA response displayed a more restricted antigen recognition profile than IgG ACPA (OR = 0.26, *P *< 0.0001).

**Conclusion:**

Our data show that several defined citrullinated antigens are recognized only by IgG ACPA, whereas others are also recognized by IgM ACPA. These observations suggest that not all citrullinated antigens are able to activate new B cells despite concurrent recognition by IgG ACPA.

## Introduction

Anti-citrullinated protein antibodies (ACPA) may be involved in the disease pathogenesis of rheumatoid arthritis (RA). ACPA can be found early in the disease course [1], even before disease onset [2], and the presence of ACPA at the time of diagnosis can predict disease course [3]. Moreover, ACPA can contribute to disease pathogenesis by activating immune cells [4,5] and the complement system [6].

The ACPA response likely represents a T-cell-dependent B-cell response, given the protein nature of the antigen recognized and the strong association with the human leukocyte antigen shared epitope alleles. The evolution of such a response is typically characterized by a first wave of IgM antibodies after the first antigen contact, quickly followed by the presence of IgG. After repeated antigen exposure, the IgG responses are further boosted while the IgM peak declines. The latter observation is explained by the presence of Ig-switched, affinity matured, memory B cell that are formed in the presence of CD4^+ ^T cells. These T cells provide the helper activity required for affinity maturation, isotype switching and memory cell formation. When such T-cell help cannot be provided, as in the case of hyper-IgM syndrome, IgG, IgA and IgE antibody levels are absent or severely reduced [7]. The presence of IgG, IgA and IgE ACPAs [5,8], therefore, provides another line of evidence for the T-cell-dependent nature of ACPA responses.

To the best of our knowledge, IgM-producing memory B cells against T-cell-dependent antigens have not been described, in contrast to T-cell-independent B-cell responses against, for example, repetitive sugar residues on bacteria [9,10]. For these reasons, it is most conceivable that the presence of IgM ACPA suggests that activation of recently recruited naïve B cells recognize citrullinated antigens because the half-life of circulating IgM is short. In this study, we hypothesized that there might be certain antigens which drive the ACPA IgM response in RA. We therefore sought to determine whether there is a difference in the fine specificity of IgG and IgM ACPA.

## Materials and methods

### Fine specificity of anticitrullinated protein antibody immunoglobulin M

We determined the fine specificity of ACPA IgM and IgG in 113 serum samples of anti-cyclic citrullinated peptide (CCP2) IgG and IgM double-positive RA patients collected from the Leiden Early Arthritis Clinic (EAC), an inception cohort of recent-onset arthritis that was initiated at the Department of Rheumatology at Leiden University Medical Center in 1993 [11]. We selected those patients who had a relatively high titer of IgM CCP2 (levels ≥ 100 AU/ml) to ensure that differences between IgG and IgM reactivity could not be explained by differences in the sensitivity associated with the detection of IgG or IgM antibodies by ELISA. For the determination of ACPA status and ACPA levels of IgM anti-CCP2, we used a commercial immunoassay kit (Euro-Diagnostica, Malmö, Sweden) with minor modifications for the detection of IgM. The collection and use of patient samples was approved by the local medical ethics committee and in compliance with the Declaration of Helsinki. All patients provided their written informed consent.

Demographic data (age, gender, disease duration and radiographic damage) of ACPA IgG-positive RA patients who had ACPA IgM ≥ 100 AU/ml were not different from patients displaying ACPA IgM levels < 100 AU/ml. The peptides used in this study are linear citrulline (Cit)-containing peptides, which are known ACPA IgG epitopes in RA, as well as their arginine counterparts [12]. Specifically, we used vimentin (Vim) 59 to 74: VYATCitSSAVCitLCitSSVP; fibrinogen-α (Fib-α) 27 to 43: FLAEGGGVCitGPRVVERH; Fib-β 36 to 52: NEEGFFSACitGHRPLDKK; and α-enolase 5 to 20: KIHACitEIFDSCitGNPTV. In addition, we tested citrullinated protein antigens (Fib and myelin basic protein (MBP)) and all of their arginine counterparts. As described previously, the presence of anti-Cit-MBP antibodies on ELISA is clinically equivalent to the original anti-Sa (Cit-Vim) on Western blot assays [13].

Fine specificity assays of ACPA IgG were performed essentially as described before [12], with minor modifications for ACPA IgM [8]. In brief, for IgM-, Cit- and arginine-containing peptides (10 μg/ml) were incubated on streptavidin-coated plates. After being washed, sera were incubated at 1:25 dilution at 37°C for 1 hour, bound antibodies were detected using rabbit anti-human IgM (Dako Denmark A/S, Glostrup, Denmark), followed by washing with horseradish peroxidase-labeled goat anti-rabbit IgG (Dako Denmark A/S). 2,2'-azino-bis(3-ethylbenzothiazoline-6-sulfonic acid) was used as a substrate, and absorbance was determined at 415 nm. In each ELISA plate, we included on the Cit-containing peptide eight representative gout controls and used these to calculate the cut-off for positivity [8]. These eight gout controls represent a set of fifty controls as established before [8] and were used on each plate to minimize plate-to-plate variation. This was defined as the mean ± 2 SD of the absorbance on the Cit-containing peptide. In addition, we verified that the difference in absorbance between wells coated with the Cit- and the arginine-containing peptide was at least 0.1 as previously described [8,12]. Therefore, sera that fulfilled both criteria were considered positive.

### Stability of the anti-citrullinated protein antibody I response

Samples from 18 patients were used to analyze the specific reactivity of IgM against CCP2, Fib-α and Fib-β over time. We used serum samples obtained at baseline and at 1, 2 and 5 years of follow-up. Samples were tested by ELISA as described above. Samples were considered positive when they displayed an absorbance value higher than the cut-off and a difference of at least 0.1 absorbance units when comparing the reactivity against the Cit-containing peptide and its arginine-containing control peptide.

### Statistical analysis

Differences between groups were analyzed using the Mann-Whitney *U *test or analysis of variance. The associations between ACPA IgM and ACPA IgG positivity were evaluated by using 2 × 2 tables for the estimation of ORs and 95% CIs. The correlation between ACPA IgM and ACPA IgG responses were determined using Spearman's rank-correlation coefficients. The χ^2 ^test was used to evaluate differences in the frequency distribution of ACPA IgG and IgM. All data were analyzed with GraphPad Prism version 4.0 software (GraphPad Software, San Diego, CA, USA) or SPSS for Windows release 16.0 software (SPSS Inc, Chicago, IL, USA). In all tests, *P *< 0.05 was considered significant.

## Results

### Anti-citrullinated protein antibody fine specificity is different from IgG fine specificity

**Figure 1:**
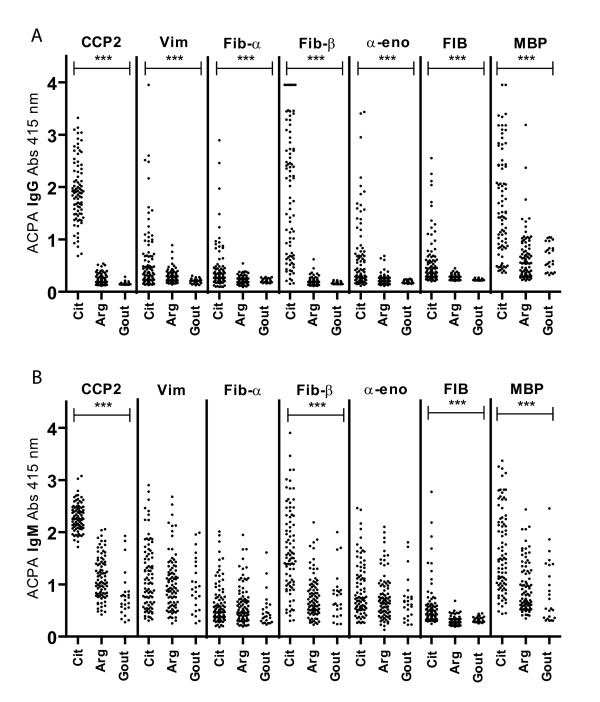
**The fine specificity of immunoglobulin G (IgG) and IgM anticitrullinated protein antibodies (ACPAs)**. The absorbance (Abs) at 415 nm of ACPA IgG **(A) **and IgM **(B) **ACPA fine specificity. ***P < 0.001. Data for gout controls from three ELISA plates are depicted. α-eno = α-enolase; Arg = arginine; CCP2 = cyclic citrullinated peptide 2; Cit = citrulline; Fib = fibrinogen; MBP = myelin basic protein; Vim = vimentin.

It has been shown that IgM ACPA can be detected in the sera of RA patients with established disease, suggesting an ongoing recruitment of new B cells into the ACPA response [8]. If IgM is the result of triggering of naïve B cells, then the fine specificity of ACPA IgM might conceivably differ from ACPA IgG, as IgM will mainly be directed against the antigens that have recently been detected by B cells [14]. Therefore, we determined the ACPA IgM and IgG responses against a set of citrullinated peptides from Fib, enolase and Vim, as well as the responses against two citrullinated proteins (Figures 1A and 1B).

Our data show that some but not all citrullinated epitopes are recognized by IgM ACPA (Table 1 and Figure 1B). These data indicate a restricted epitope recognition profile by IgM ACPA, as some epitopes were not recognized by IgM in all analyzed patient sera.

**Table 1:** Specific positivity of anticitrullinated protein antibody immunoglobulin G and immunoglobulin M in 113 rheumatoid arthritis patients directed against a set of defined specificities

Epitope	IgG (%)	IgM (%)
CCP2	100	100
Vim	56	7
Fib-α	33	4
Fib-β	94	40
α-enolase	55	8
Fib	53	40
MBP	63	40

CCP2 = cyclic citrullinated peptide 2; Fib = fibrinogen; IgG = immunoglobulin G; IgM = immunoglobulin M; MBP = myelin basic protein; Vim = vimentin.

**Figure 2:**
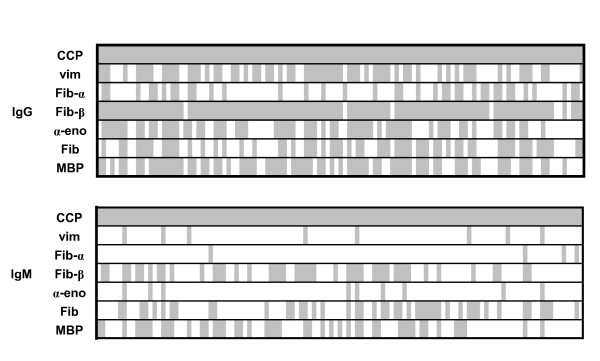
**Positivity of immunoglobulin G (IgG) and IgM anticitrullinated protein antibodies (ACPAs)**. The citrullinated epitopes recognized by IgG and IgM ACPAs in each individual. The gray area represents positive reactivity, and the white area represents lack of reactivity. α-eno = α-enolase; CCP = cyclic citrullinated peptide; Fib = fibrinogen; MBP = myelin basic protein; Vim = vimentin.

We next determined the recognition profile of IgG ACPA. Although not all patient sera recognized all citrullinated epitopes, the IgG ACPA epitope recognition pattern was clearly much broader than the epitope recognition profile of IgM ACPA (Figures 1A and 2). In fact, when all ACPA reactivities were analyzed as a group, the chance of having an IgM-positive response was four times lower compared to IgG (OR = 0.26, *P *< 0.0001). For those reactivities where, next to IgG ACPA, IgM ACPA responses also were apparent, a correlation between the titers of IgG and IgM ACPAs was observed between the positive samples (data not shown).

When reactivity patterns were directly compared at an individual level, we observed that IgM ACPAs against some citrullinated epitopes, but not others, were present. However, in all cases, IgM positivity was paralleled by the presence of IgG recognizing the same epitopes. In contrast, patients can display certain IgG ACPA reactivities in the absence of IgM reactivities against the same antigen (Figure 2). Together, these data indicate that IgM ACPAs display a more restricted antigen recognition profiles as compared to IgG ACPAs.

### Stability of the anti-citrullinated protein antibody I response

**Figure 3:**
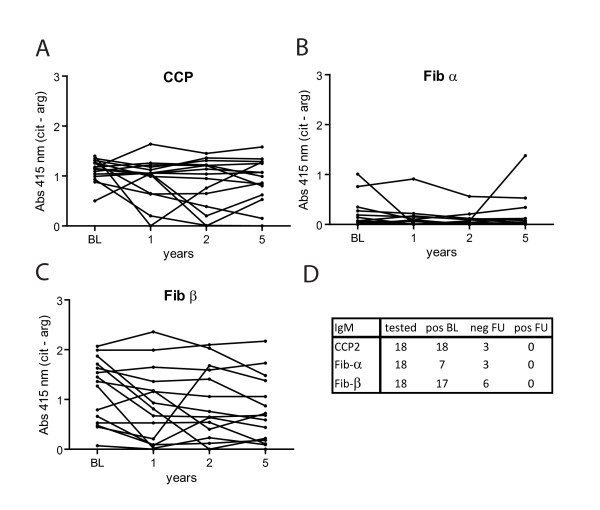
**Stability of the immunoglobulin M anticitrullinated protein antibody response**. Sera taken from 18 patients at baseline (BL) and at 1, 2 and 5 years of follow-up were analyzed by ELISA for reactivity against cyclic citrullinated peptide 2 (CCP2), fibrinogen (Fib)-α and Fib-β. **(A) **through **(C) **Graphs displaying the immunoglobulin M (IgM) reactivity to the indicated peptides over time. The absorbance of citrulline (cit) reactivity minus absorbance of the arginine (arg) reactivity is shown. **(D) **Table showing the number of positive patients at baseline (BL) as well as the conversion toward negative or positive during one of the follow-up (FU) time points analyzed.

To address the question whether the observed differences between IgG and IgM reactivity are limited to baseline samples only or whether they are also present at later follow-up, we analyzed ACPA IgM reactivity against three peptides over time. The reactivity of ACPA IgM against CCP2, Fib-α and Fib-β at baseline and at 1, 2 and 5 years of follow-up was analyzed in sera of 18 patients. We observed that the ACPA IgM levels against these peptides appeared rather stable (Figures 3A to 3C). Some patients whose sera reacted with CCP2, Fib-α and Fib-β at baseline may over time become IgM-negative (Figure 3D). Importantly, none of the patients tested became IgM-seropositive against either Fib-α or Fib-β peptides. These data indicate that the observations made using the baseline samples (Figures 1 and 2) would also be made at any of the follow-up time points. Collectively, these data therefore indicate that the ACPA IgM response is narrower than the ACPA IgG response, an observation not applicable only to the baseline results but also during follow-up.

## Discussion

In this study, we observed that ACPA IgM responses are different from ACPA IgG responses, as they display a more restricted antigen recognition pattern. These data are intriguing, as they indicate that the regulation of the IgM ACPA response differs from the regulation of B cells producing IgG-directed against citrullinated antigens. Although the reason for this difference is not known, we think it is most conceivable that these findings are explained by a limited recruitment of new B cells into the ACPA response that is driven by some, but not other, citrullinated antigens. Given the short half-life of circulating IgM and the lack of memory B cells producing IgM against protein antigens, we think that the IgM ACPAs detected in this study are produced by new B cells that are recruited into the ACPA response against certain citrullinated antigens. Even in the case where ACPA IgM-producing memory B cells exist, it is still interesting that such cells are present only against certain citrullinated antigens.

For this study, we selected patients who are double-positive for IgG and IgM CCP2 reactivity. To exclude the possibility that differences between IgG and IgM may be explained by differences in the sensitivity associated with the detection of IgG or IgM antibodies by ELISA, we selected only patients with an IgM level of at least 100 AU/ml. Indeed, in the setup we used, we observed that, although the IgG responses against the different citrullinated antigens were rather similar in absorbance units, IgM reactivity was detected only against some antigens but not others. This observation was made at baseline but appears also to be present at follow-up. To ensure that we did not introduce additional bias into our data, we compared the demographic data (age, gender, disease duration and radiographic damage) of ACPA IgG-positive RA patients who had ACPA IgM ≥ 100 AU/ml with those who had ACPA IgM < 100 AU/ml and observed no differences.

Since IgM rheumatoid factor (RF) could also be a confounding factor, we compared RF levels between the patients who displayed ACPA IgM ≥ 100 AU/ml with those who had ACPA IgM < 100 AU/ml and observed that in RA patients with ACPA IgM ≥ 100 AU/ml, the levels of RF IgM were higher than in RA patients who had ACPA IgM < 100 AU/ml (RF IgM 50 AU/ml (25 to 118) and 28 AU/ml (12 to 60), respectively). To exclude the possibility that our findings were influenced by IgM RF, we analyzed ACPA IgM specificities in relation to IgM RF. We observed that IgM RF-positive samples can be negative for IgM ACPA reactivities and that, in the absence of IgM RF, IgM ACPA reactivities can be detected readily (data not shown). RF-positive samples that have IgG reactivity against all fine specificity epitopes may have IgM ACPA against only some antigens and not others, confirming that our assay did not merely detect IgM RF. Previously, we addressed this issue experimentally [8]. When RFs were depleted from RF IgM-positive, ACPA IgM-positive and ACPA IgG-positive sera using IgG-coated Cyanogen bromide*-*activated-Sepharose beads (Sigma-Aldrich, St Louis, MO, USA), there was no reduction of ACPA IgM levels. Moreover, after mixing sera which were highly positive for RF IgM but negative for ACPA IgG and ACPA IgM with sera which were RF IgM- and ACPA IgM-negative but ACPA IgG-positive, ACPA IgM could not be detected [8]. Collectively, these observations support the notion that true IgM ACPA and not IgM RF bound to ACPA IgG were detected by the methods employed.

Although it is tempting to speculate, these studies should not be taken as an argument for the involvement of the antigens analyzed here in the recruitment of new B cells into the ACPA response. Peptides are unlikely to reflect correctly the three-dimensional structure of citrullinated proteins that form the epitope for antibodies. Moreover, ACPA IgG is cross-reactive to multiple citrullinated antigens [15,16], and therefore recognition of a citrullinated antigen by ACPA IgG does not indicate that this antigen is necessarily involved in the induction of B-cell responses. Nonetheless, our data do show that the IgM ACPA response is significantly more restricted than that of the IgG ACPA present in the same patient.

How IgM can be formed in the presence of an active IgG response against the same antigen is not clear. In other situations, as exemplified by the prophylactic administration of anti-Rhesus D antigen antibodies to pregnant women carrying a Rhesus D-positive child, the presence of IgG against a certain antigen will prevent the induction of a novel IgM response. The mechanism behind this protective measure is thought to be mediated by either the capture and clearance of circulating Rhesus D antigen and/or by IgG-Rhesus D immune complexes that inactivate new Rhesus D-reactive B cells through FcγRIIB, the inhibitory Fcγ IIB receptor [17]. Clearly, IgG ACPAs do not inhibit the activation of IgM-positive, citrullinated antigen-reactive B cells. The reason for this finding is not known but could possibly be explained by the low avidity of the ACPA [18], conceivably resulting in "nonstable" immune complexes unable to trigger FcγRIIB.

## Conclusions

Collectively, our data show that the immune response against one citrullinated antigen is different from the immune response against another citrullinated protein. Some responses are dominated by IgG, whereas both IgM and IgG responses were found for other ACPA antigens. Elucidation of the mechanism behind this observation could be of relevance to the identification of those citrullinated antigens that drive ACPA responses and could provide clues to how the continuous recruitment of new B cells can be halted.

## Abbreviations

ACPA: anticitrullinated protein antibody; Arg: arginine; AU: arbitrary unit; BL: baseline; Cit: citrulline; CCP2: cyclic citrullinated peptide 2; EAC: Early Arthritis Clinic; ELISA: enzyme-linked immunosorbent assay; Fib: fibrinogen; FU: follow-up; Ig: immunoglobulin; IgM: immunoglobin M LUMC: Leiden University Medical Center; MBP: myelin basic protein; RA: rheumatoid arthritis; RF: rheumatoid factor.

## Competing interests

The authors declare that they have no competing interests.

## Authors' contributions

PS, TWJH, REMT and LAT conceived the study and participated in its design and edited the manuscript. PS and LAT drafted and prepared the manuscript and performed the statistical analyses. AW assisted with manuscript preparation. PS, LVT, GSR and NL performed blood analyses. JWD provided peptides essential for the assays. All authors contributed to the interpretation of the study findings and approved the final manuscript.
